# Seed Weight and Genotype Influence the Total Oil Content and Fatty Acid Composition of Peanut Seeds

**DOI:** 10.3390/foods11213463

**Published:** 2022-11-01

**Authors:** Weilan Li, Eunae Yoo, SooKyeong Lee, Jungsook Sung, Hyung Jun Noh, So Jeong Hwang, Kebede Taye Desta, Gi-An Lee

**Affiliations:** 1 National Agrobiodiversity Center, National Institute of Agricultural Sciences, Rural Development Administration, Jeonju 54874, Korea; 2 International Technology Cooperation Center, Rural Development Administration, Jeonju 54875, Korea; 3 Department of Applied Chemistry, Adama Science and Technology University, Adama 1888, Ethiopia

**Keywords:** food, fatty acid, peanut, seed, seed weight, oil

## Abstract

Peanut, an important oilseed crop cultivated worldwide as a dietary food, is a good food source with health benefits. To explore the potential benefits of peanuts as a food resource, 301 peanut accessions were evaluated to determine the effect of seed weight and genotype on total oil content and fatty acid composition. Total oil was extracted using the Soxhlet method and fatty acids were analyzed by gas chromatography mass spectrometry. Wide variations in the 100-seed weight, total oil content, and fatty acid profile were observed among genotypes and accession types. An effect of seed weight on the fatty acid composition of peanut seeds was observed. Increases in the oleic acid content and decreases in the linoleic acid content occurred in association with increases in the 100-seed weight. Moreover, the 100-seed weight, total oil content, and individual and total fatty acid contents, except arachidic acid, differed significantly (*p* < 0.001 or 0.05) among the accession types of landrace, cultivar, breeding line, and unknown. The discovery of this high diversity could contribute to further studies of peanut domestication and evolutionary classification. Our findings are important for the selection of peanut seeds with health benefits and development of new varieties of peanut with health benefits.

## 1. Introduction

Peanut (*Arachis hypogaea* L.), which is widely cultivated in the tropics and subtropics, is an economically important oilseed crop used for vegetable oil production. Peanut production has increased every year because peanuts are used for the popular vegetable oil, are valued as food, and are used in the medicine industry. According to the Food and Agriculture Organization (FAO) of the United Nations, from 1970 to 2010, world peanut production increased from 16,719,000 metric tons (MT) to 34,968,000 MT, and rose to 48,760,000 MT in 2019. China is the main producer of peanut, and accounts for nearly half of the world production, followed by India, Nigeria, and USA. The great increase of peanut production contributes a lot to the food supply and other benefits.

As a functional food, peanut seed contains oil (47–50%) and protein (27–29%), along with various nutrients beneficial to health such as minerals, vitamin E, folic acid, niacin, antioxidants, and biologically active polyphenolics, including flavonoids and isoflavones [1,2,3]. The total unsaturated fatty acid and total saturated fatty acid contents of peanut oil are about 80% and 20%, respectively. Around 50% of unsaturated fatty acid is monounsaturated fatty acid (oleic acid, C18:1), 30% is polyunsaturated fatty acid (linoleic acid, C18:2), and the linolenic acid (C18:3) was almost undetectable. Almost 10% of saturated fatty acid is palmitic acid (C16:0), and a further 10% is stearic acid (C18:0), arachidic acid (C20:0), gondoic acid (C20:1), behenic acid (C22:0), lignoceric acids (C24:0), and so on [4,5,6]. The flavor, quality, and shelf-life of peanut seeds and products are strongly related to the lipid chemistry, especially fatty acid composition [3,7]. A high ratio of oleic acid to linoleic acid results in high-quality seeds and related products with a long shelf-life, and could also confer health benefits, thereby improving the marketability of peanuts [8]. Oleic acid reduces systolic blood pressure, which decreases the risk of cardiovascular disease [9,10]. An oleic acid-rich diet also helps reduce the level of blood glucose and increases the high-density lipoprotein (HDL) to low-density lipoprotein (LDL) ratio, which also has health benefits [11,12]. As a result, peanut seeds with a higher level of oleic acid are more popular with consumers; the development of new varieties that contain high levels of oleic acid is therefore important.

Environmental and genetic factors control the types and amounts of metabolites in plants. Environmental factors such as climate, planting year and date, growth location, temperature, and drought stress can affect the agricultural traits, oil content and fatty acid composition of peanut seeds [13,14,15,16,17,18]. Genetic factors play a key role in the diversity of metabolites and phenotypic characteristics. Various strategies have been applied to explore the potential genetic resources, and improve the nutritional quality, of peanut seeds and its products [3,19,20,21,22]. Peanuts have been improved over time in terms of desired traits through breeding programs. Landraces, varieties selected by farmers for adaptation to local environments and food supply, are an important source of agricultural biodiversity, which provide useful traits for breeding programs [23]. Breeding line is a group of identical pure-breeding organisms which contain unique phenotypes and genotypes. Cultivar is a type of plant that have been bred for desired traits, which are reproduced in each new generation. Over the years, landraces have been artificially selected through domestication and breeding to meet consumer demand. Consequently, a wide variation of accession types of landraces, cultivars, and breeding lines have been generated [24,25,26,27].

To ensure health benefits from consuming peanuts, many researchers have investigated the factors that control the agronomical traits, total oil content, and fatty acid profile of various peanut genotypes [5,28,29,30,31,32,33]. Peanut seed traits, including the size, shape, weight, yield, and seed coat color, have been well studied [32,33]. As an important component of seed yield, seed weight has attracted much attention. Seed weight has been investigated at the molecular level using quantitative trait loci (QTL), and genes regulating seed weight have been identified [21,22]. Differentially expressed genes during development could also affect the yield, metabolites, and quality of seeds. Therefore, studying the associations between seed weight and metabolites is necessary to identify peanut seeds with health benefits, and to obtain information that could aid the development of new high-quality peanut varieties. However, our understanding of the correlations among seed weight, oil content, and fatty acid profiles is limited. Research on a large number of peanut genotypes is required to obtain a thorough understanding of the associations among these factors.

The objectives of this study were to investigate the influence of seed weight and genotype on total oil content and fatty acid composition in peanut seeds, finding new information which could aid the identification of peanut seeds with health benefits and the development of new high-quality peanut varieties. As a result, 301 peanut genotypes in cultivar, breeding line, landrace, and unknown categories were collected and cultivated. The 100-seed weight, total oil content, and fatty acids (arachidic acid, linoleic acid, oleic acid, palmitic acid, stearic acid) were analyzed. Analysis of variance (ANOVA), Pearson correlation, principal component analysis (PCA), and hierarchical clustering analysis (HCA) were applied for the data analysis. Variations in 100-seed weight, total oil content, and fatty acid profile among genotypes were studied. The effects of seed weight and genotype on total oil content and fatty acid composition were also investigated. Our findings could provide important knowledge for peanut research, including domestication studies, selection of seeds with health benefits, and development of new peanut varieties with high levels of oleic acid.

## 2. Materials and Methods

### 2.1. Reagents and Chemicals

The chemicals and reagents used in this study, including anhydrous sodium sulfate, *n*-hexane, sodium hydroxide, 14% boron trifluoride-methanol (BF_3_-methanol), and fatty acid standards (palmitic acid, stearic acid, oleic acid, linoleic acid, and arachidic aid), chloroform, methanol, HPLC analytical grade water were purchased from Sigma Aldrich (St. Louis, MO, USA). All chemicals were of analytical grade and used without further purification.

### 2.2. Peanut Cultivation and Sample Preparation

Seeds of 301 peanut accessions with different genotypes (cultivar, breeding line, landrace, and unknown) were obtained from the gene bank of the National Agrobiodiversity Center (NAC), Rural Development Administration (RDA), Jeonju, South Korea. The peanut seeds were planted and cultivated in the National Institute of Crop Science (NICS) experimental field (35°29′33.2″ N, 128°44′35.7″ E) in 2018. The seeds were harvested at full maturity from September to October, and dried in a VS-1202D drying oven (Vision Scientific, Bucheon, Korea) for 3 days at 50 °C. For each accession, the 100-seed dry weight was measured and recorded. The dried seeds of the peanut germplasms were ground into a fine powder using an electronic grinder and sieved through a 315 µm screen, and then stored in a plastic bag at −20 °C prior to the analysis.

### 2.3. Determination of Total Oil Content

The total oil content was determined using a modified version of a previously described method [34]. For oil extraction, the Soxhlet apparatus was used (SoxtecTM 2043 system; FOSS Tecator AB, Hillerod, Denmark). In brief, 1 g of seed powder was mixed with 50 mL of *n*-hexane and loaded onto the extraction system set at 135 °C and the boiling, rinsing, and recovering times were 30, 60, and 30 min, respectively. Following extraction, the oil was cooled to room temperature and weighed. The total oil content was calculated as the percentage weight of oil obtained relative to the weight of extracted seed sample (three measurements).

### 2.4. Analysis of Fatty Acids

The fatty acids were analyzed using a previously described method, with some modifications [35]. The sample and standards were initially derivatized. To accomplish this, each tube containing crude fat was treated with 2 mL of 0.5 M NaOH to transmethylate the fatty acids, and then vortexed for 5 s before heating for 10 min at 80 °C in a water bath and cooling at room temperature. We added 2 mL of 14% cold boron trifluoride-methanol to each tube, followed by vortexing for 5 s before incubation at 80 °C in a water bath for 10 min, cooling to room temperature. Finally, 7 mL of *n*-hexane and 2 mL of H_2_O were added, followed by vortexing for 10 s, and centrifugation for 10 min at 4 °C and 3000 rpm. The *n*-hexane (upper liquid layer) was collected and filtered using filter paper where anhydrous sodium sulfate powder was placed on top of it, and the filtrate was transferred to sample vials for gas chromatography and stored at −20 °C until analysis.

The fatty acid methyl esters (FAMEs) were analyzed using the GCMS-QP2010 UltraGas Chromatography instrument (Shimadzu Co., Kyoto, Japan) equipped with a 19091N-136 INNOWAX column (0.25 mm × 60 m, 0.25 mm; Agilent Technologies Inc., Santa Clara, CA, USA). During analysis, the column was initiated at 150 °C, increased to 200 °C at a rate of 4 °C/min, and then maintained at 220 °C for 5 min. The temperature of the injector and detector were set at 250 °C and 300 °C, respectively. The injection volume was 10 µL and nitrogen was used as a carrier gas (N_2_), at a flow rate of 0.6 mL/min. The fatty acids were identified using the related standards and quantified as the percentage of total fatty acid using the peak areas.

### 2.5. Statistical Analysis

All experiments were conducted in triplicate. The total oil content and individual and total fatty acid contents were recorded as the mean ± standard deviation (SD). ANOVA, Pearson correlation, PCA, and HCA were performed using R software (version 4.1.2; RStudio, Boston, MA, USA).

## 3. Results and Discussion

### 3.1. The 100-Seed Weight, Total Oil Content, and Fatty Acid Profile of 301 Peanut Accessions

The 100-seed weight, total oil content, and fatty acid data are presented in Table 1. A wide range of values were observed for each parameter. The 100-seed weight ranged from 27.61 to 124.94 g, with a mean value of 64.17 g. Similar to previous studies, peanut seed weight varied widely among the genotypes [3,22]. The total oil content was in the range of 44.59–64.55%. There were also large variations in the contents of individual fatty acids, including palmitic acid (7.41–14.06%), stearic acid (1.78–7.22%), arachidic acid (0.93–2.42%), oleic acid (40.17–63.35%), and linoleic acid (21.66–44.49%). Compared to previous studies, wider variations in the parameters were seen in this study [6,17,36], which could be attributed to the large number of genotypes used in this study. As reported in other studies, oleic acid and linoleic acid were the main fatty acids, followed by palmitic acid. These three fatty acids accounted for almost 90% of the oil [5,37,38]. The variation of oleic acid to linoleic acid ratio was in the range of 0.90–2.87, with a mean of 1.58. Unlike our findings, a previous study of traditional Indonesian peanuts reported lower levels of oleic acid and linoleic acid, and a lower oleic acid to linoleic acid ratio (29.56–47.61 g/100 g, 27.07–46.84 g/100 g, and 0.65–1.34, respectively) [32]. In contrast, US studies reported improved peanut genotypes with high levels of oleic acid [6,17,36]. For example, the runner-type peanut cultivars contain oleic acid and linoleic acid in the ranges of 44.78–82.17% and 2.85–33.92%, respectively [6]. The oleic acid to linoleic acid ratio is an important parameter when evaluating food quality and security in the context of human health. Although runner-type peanut cultivars produce seeds with a high oleic acid content, they have a very long growing period [18,39]. Consequently, it is essential to obtain valuable genetic information for the development of new peanut varieties with a high level of oleic acid and pleasing agricultural traits.

### 3.2. Total Oil Content and Fatty Acid Profile: Associations with Seed Weight

**Figure 1:**
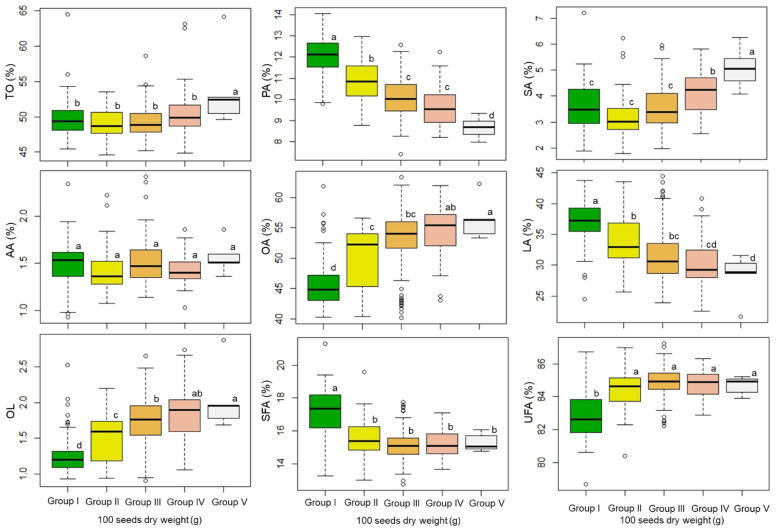
Total oil content, and individual and total fatty acid contents according to 100-seed weight groups. Group I, Group II, Group III, Group IV, and Group V represent the genotypes have a 100-SW of 25–45 g, 45–65 g, 65–85 g, 85–105 g, and 105–125 g, respectively. TO = total oil, PA = palmitic acid, SA = stearic acid, OA = oleic acid, LA = linoleic acid, AA = arachidic acid, OL = oleic acid to linoleic acid ratio, SFA = total saturated fatty acid, UFA = total unsaturated fatty acid. Different letters indicate significant differences between groups (*p* < 0.05).

The influence of seed weight on the total oil content, and individual and total fatty acid contents, were investigated. The peanut genotypes were classified into five groups (Group I, 25–45 g; Group II, 45–65 g; Group III, 65–85 g; Group IV, 85–105 g; Group V, 105–125 g) based on their 100-seed weight, and the total oil content and fatty acid contents of each group were analyzed. As shown in Figure 1 and Table S1, the highest total oil content was that of Group V, and there was no significant difference in total oil content among the groups with a 100-seed weight <105 g. The highest stearic acid content was that of Group V, followed by Group IV. Nevertheless, seed weight did not bring a significant difference in stearic acid content among the groups with a 100-seed weight lower than 85 g. Additionally, there were no significant differences in arachidic acid content among the different seed weight groups. Group I had a significantly higher total saturated fatty acid content (17.07%) and significantly lower total unsaturated fatty acid content (82.93%) than the other groups. Interestingly, the oleic acid content and oleic acid to linoleic acid ratio increased with an increase in the 100-seed weight, and both showed significant differences among the 100-seed weight groups (Group V > Group IV > Group III > Group II > Group I). In contrast, the linoleic acid and palmitic acid contents decreased with an increase in the 100-seed weight (Group I > Group II > Group III > Group IV > Group V). Several studies have investigated peanut agricultural traits, including plants and seeds, and the oil metabolites and fatty acid profile of seeds [5,28,29,30,31,32,33]. However, few studies have considered the associations among seed weight, oil content, and fatty acid profile. Klevorn et al. (2016) investigated the oleic and linoleic acid contents according to seed fresh weight at different seed developmental stages; an increase in oleic acid content and decease in linoleic acid content were associated with an increase in seed fresh weight as seed growth progressed [40]. Moreover, Wang et al. (2018) investigated eight peanut breeding lines and observed non-significant and weak correlations between 100-seed weight and fatty acid composition [3]. These noteworthy findings provided useful information for peanut breeding programs; however, only a few genotypes were used, and the study concentrated on changes at different seed development stages. To obtain a better understanding of such associations, a comprehensive study performing multivariate analysis on many genotypes will be needed. In the present study, 301 peanut genotypes in different categories were investigated in terms of their 100-seed weight, total oil content, and fatty acid profiles. Our findings strongly suggested that peanut seed weight influenced the fatty acid composition of seeds, in particular, the increase in oleic acid content and decrease in linoleic acid content were closely associated with an increase in the 100-seed weight. Such associations should be considered as a good parameter for the selection of high-quality peanut seeds, and the development of new varieties containing high levels of oleic acid in the breeding program.

### 3.3. Variations of Seed Weight, Total Oil Content, and Fatty Acids among Accession Types

The 100-seed weight, total oil content, and individual and total fatty acids of the 301 peanut genotypes are summarized in Table 2 according to their accession types. Wide variations of 100-seed weight were observed in the cultivar, breeding line, landrace, and unknown genotypes, which ranged from 39.92 to 106.44, 27.61 to 124.94, 34.56 to 92.26, and 27.79 to 83.38 g, respectively. In each of the accession types, the major fatty acids were oleic acid and linoleic acid, in agreement with previous studies [32,37]. Moreover, 100-seed weight, total oil content, and individual and total fatty acids, except arachidic acid, differed significantly (*p* < 0.001 or 0.05) among accession types. The 100-seed weight, stearic acid content, oleic acid content, and oleic acid to linoleic acid ratio decreased in the order of cultivar > breeding line > landrace > unknown genotype, whereas the linoleic acid and palmitic acid contents increased in the order of cultivar < breeding line < landrace < unknown genotype. Landraces, which are key sources of naturally evolved nutrients, exhibit major genetic variability. Due to their adaptation to the environment, landraces are used for the selection of desired traits in breeding strategies, and the improved cultivars and breeding lines are richer in selected metabolites than landraces [27,41]. The differences among peanut accession types suggested that the diversity of 100-seed weight may be associated with peanut domestication [42,43]. The metabolite differences reflected the large variability in genotypic background. A previous study reported that oil composition was associated with the domestication level [44]. The wide variation observed in this study, especially in fatty acid composition, could aid the evolutionary classification of peanuts.

### 3.4. Pearson Correlation Analysis

Pearson correlation analysis of the 100-seed weight, total oil content, and fatty acid profile was conducted; the correlation coefficients (r) and levels of significance are given in Table 3. Oleic acid was negatively correlated with linoleic acid (r = −0.98, *p* < 0.001) and palmitic acid (r = −0.80, *p* < 0.001). Shin et al. (2010) documented an inverse association of oleic acid with linoleic acid (r = −0.997, *p* < 0.001) and palmitic acid (r = −0.971, *p* < 0.001) in a study of runner-type peanuts [6]. In a study of Indian peanut genotypes, Nawade et al. (2016) recorded similar associations of oleic acid and linoleic acid (r = −0.96, *p* < 0.01), and oleic acid and palmitic acid (r = −0.87, *p* < 0.01) [45]. Likewise, Andersen and Gorbet (2002) noted that oleic acid was inversely related to linoleic acid (r = −0.996, *p* < 0.0001) and palmitic acid (r = −0.959, *p* < 0.0001) [17]. The negative correlation between oleic acid and linoleic acid could be explained by the function of ∆^12^-Fatty acid desaturase (FAD), which converts oleic acid to linoleic acid [46]. Remarkably, the 100-seed weight was strongly associated with the metabolites in peanut seed. For example, the 100-seed weight had significant positive correlations (*p* < 0.001) with oleic acid (r = 0.60), stearic acid (r = 0.25), total unsaturated fatty acid (r = 0.51), and the oleic acid to linoleic acid ratio (r = 0.58). In contrast, significant negative correlations (*p* < 0.001) were observed between the 100-seed weight and palmitic acid (r = −0.71), linoleic acid (r = −0.55), and total saturated fatty acid (r = −0.51). Wang et al. (2018) observed weak and non-significant correlations between the 100-seed weight and fatty acids (oleic acid, linoleic acid, stearic acid, and palmitic acid) in eight peanut breeding lines [3]. These differences between studies could be due to the large number of genotypes used in the present study, which conducted more comprehensive analyses and comparisons.

Additionally, the correlations between 100-seed weight, total oil content, and fatty acid profile were evaluated for the individual accession types in this study (Figure S2 and Table S2). Interestingly, the associations obtained in different accession types evidenced similar trends and were in accordance with those obtained via analysis of the whole dataset. For example, there was an inverse correlation between oleic acid and linoleic acid for all accession types, i.e., for cultivar (r = −0.94, *p* < 0.001), breeding line (r = −0.97, *p* < 0.001), landrace (r = −0.98, *p* < 0.001), and unknown (r = −0.97, *p* < 0.001). Notably, the 100-seed weight was positively correlated with oleic acid content in the cultivar (r = 0.63, *p* < 0.01), breeding line (r = 0.48, *p* < 0.001), landrace (r = 0.61, *p* < 0.001), and unknown (r = 0.39, *p* < 0.001) genotypes. Moreover, the associations of seed weight with linoleic acid and palmitic acid were significant and negative. These results provide additional evidence corroborating the notion that seed weight affects the fatty acid composition.

### 3.5. PCA Results

**Figure 2:**
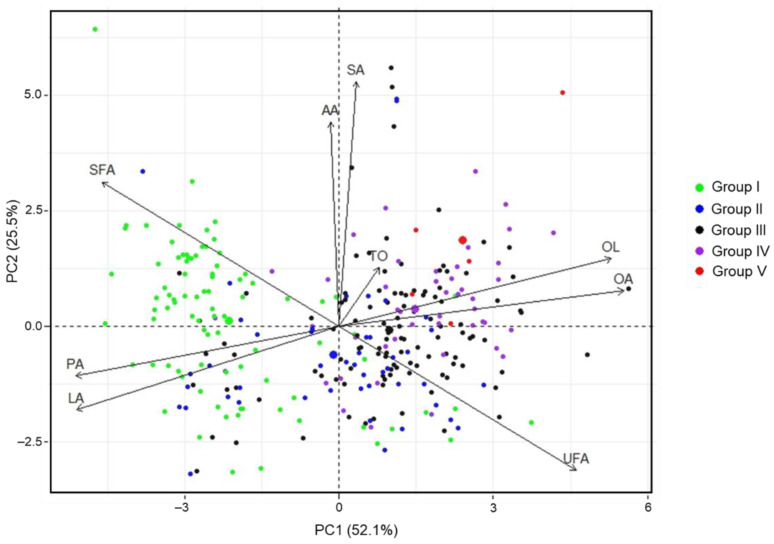
Principal component biplot for peanut accessions of the total oil content, and individual and total fatty acid contents, based on the entire dataset. Group I, Group II, Group III, Group IV, and Group V represent the genotypes have a 100-SW of 25–45 g, 45–65 g, 65–85 g, 85–105 g, and 105–125 g, respectively. TO = total oil, PA = palmitic acid, SA = stearic acid, OA = oleic acid, LA = linoleic acid, AA = arachidic acid, OL = oleic acid to linoleic acid ratio, SFA = total saturated fatty acid, UFA = total unsaturated fatty acid.

A PCA of the whole data set (301 accessions) was conducted to assess the metabolic responses to seed weight. Nine principal components (PCs) were obtained, and the related parameters of the first five PCs are summarized in Table S3. The eigenvalues of PC1 (4.69) and PC2 (2.30) were >1 and explained 77.62% of the total variance (52.09% and 25.53%, respectively). The remaining seven PCs (PC3–PC9) had progressively smaller eigenvalues (0.98, 0.65, 0.37, 0.02, 0, 0, and 0, respectively). Hence, PC1 and PC2 were used to construct score and loading plots for the analysis of distributions and associations among the total oil content and fatty acid profiles of peanut genotypes. Palmitic acid (16.97%), oleic acid (19.86%), linoleic acid (16.93%), the oleic acid to linoleic acid ratio (18.17%), total saturated fatty acid (13.78%), and total unsaturated fatty acid (13.79%) made the largest contributions to the variance along PC1. Stearic acid (36.97%) and arachidic acid (25.81%) were the main contributors to the variance along PC2. Score and loading plots showed the distribution and association of total oil content and fatty acids (Figure 2). Genotypes with a heavy 100-seed weight had higher levels of oleic acid, and higher oleic acid to linoleic acid ratios, but they also had lower levels of palmitic acid and linoleic acid. Most of the genetic resources of Group I were on the negative side of PC1, whereas most of the accessions with a 100-seed weight >45 g were on the positive side of PC1. All genotypes of Group V were on the positive sides of PC1 and PC2. Furthermore, distinct aggregations of the genotypes of Groups I and III, I and IV, I and V, and II and V were observed (Figure 2 and Figure S1). The associations between variables were visualized by PCA. The correlation between two variables was defined by their angle; a positive correlation corresponds to an angle <90° [47]. In the present study, PCA revealed similarities and differences of target metabolites, and the correlation coefficients between variables, and successfully segregated the genotypes of the different 100-seed weight groups. The associations of variables revealed by PCA were consistent with results derived from Pearson correlation analysis (Table 3). The dissimilar values of the variables in the 100-seed weight groups obtained by ANOVA (Figure 1) also supported the separation of different 100-seed weight groups in PCA. In an earlier study, Brown et al. (1975) used PCA to determine the influences of variety and growing conditions on the fatty acid composition of peanut [48]. In another study, Shin et al. (2010) applied a chemometric approach by PCA for the fatty acid profile of peanut, and successfully distinguished normal-, mid-, and high-oleic acid peanut genotypes [6]. Correlations between fatty acid variables like those in previous studies were observed in the current study. Our study revealed the effect of seed weight on fatty acid composition through a comprehensive multivariate analysis of a large number of peanut genotypes.

Moreover, the associations and contributions of target metabolites of different 100-seed weight groups were subjected to PCA, and loading plots were generated (Figure S3). The variance of PC1 and PC2 for Groups I–V were 50.8% and 22.0% (Figure S3A), 48.1% and 32.8% (Figure S3B), 45.5% and 29.5% (Figure S3C), 49.5% and 26.4% (Figure S3D), and 83.8% and 12.1% (Figure S3E), respectively. The oleic acid content was closely related to the oleic acid to linoleic acid ratio and was negatively correlated with the linoleic acid and palmitic acid contents, irrespective of seed weight. Oleic acid was weakly associated with the other individual fatty acids, regardless of seed weight difference. The loadings of oleic acid and the oleic acid to linoleic acid ratio were on the negative side of PC1 in Group I but contributed to the positive side of PC1 in other groups. The arachidic acid and stearic acid contents were closely associated with each other, and the loadings in Group V were on the negative side of PC2, whereas they were on the positive side of PC2 in the other groups. The loadings of total oil content changed from the negative to positive side of PC1 for accessions with a 100-seed weight >65 g. Additionally, the associations between total oil content and the other variables transitioned from weak to strong for oleic acid, stearic acid, and arachidic acid for accessions with a 100-seed weight >105 g. In conclusion, the fatty acid profiles of peanut seeds were influenced by seed weight; the associations and loadings of variables differed according to 100-seed weight.

### 3.6. The HCA Results

**Figure 3:**
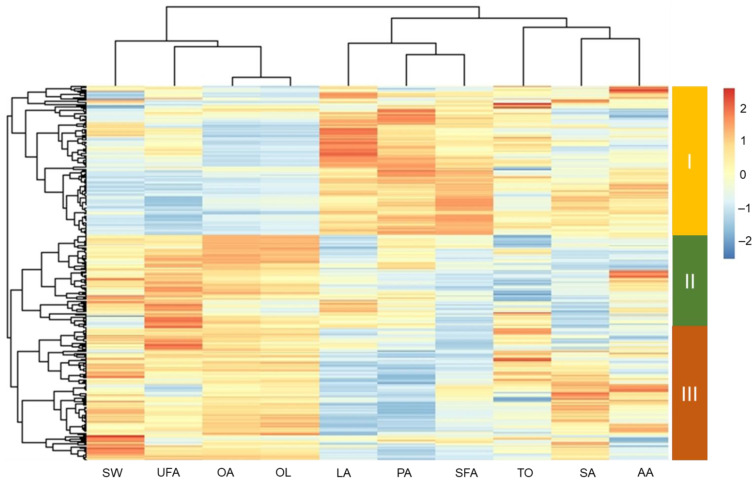
Hierarchical cluster analysis of seed weight, total oil content, and individual and total fatty acid contents using the whole dataset. SW = 100-seed weight, TO = total oil, PA = palmitic acid, SA = stearic acid, OA = oleic acid, LA = linoleic acid, AA = arachidic acid, OL = oleic acid to linoleic acid ratio, SFA = total saturated fatty acid, UFA = total unsaturated fatty acid.

A hierarchical cluster analysis was conducted of the 100-seed weight, total oil content, and fatty acid profile using the whole dataset (Figure 3 and Table S4). A total of 301 accessions were classified into three clusters; the related peanut genotypes used in this study are listed in Table S5. The 100-seed weight, total oil content, oleic acid to linoleic acid ratio, and palmitic acid, stearic acid, oleic acid, linoleic acid, total saturated fatty acid, and total unsaturated fatty acid contents differed significantly among the three clusters, but there was no significant difference in arachidic acid content. Cluster I consisted of 120 accessions, and had the highest levels of palmitic acid, linoleic acid, and total saturated fatty acid. Cluster II contained 75 accessions and had the highest levels of total unsaturated fatty acid. Cluster III had 106 accessions and the highest 100-seed weight, total oil content, oleic acid to linoleic acid ratio, and stearic acid, arachidic acid, and oleic acid contents. Intriguingly, the 100-seed weight, oleic acid content, and oleic acid to linoleic acid ratio increased in the order of cluster I < cluster II < cluster III, while the linoleic acid and palmitic acid contents decreased in the order of cluster I > cluster II > cluster III. These relationships suggest that genotypes with a higher 100-seed weight may contain higher levels of oleic acid and lower levels of linoleic acid.

## 4. Conclusions

In this study, 301 peanut genotypes were evaluated in terms of 100-seed weight, total oil content, and fatty acid profile. Wide variations were observed in all these characteristics. The results showed the influence of 100-seed weight and genotype on total oil content and fatty acid composition. The 100-seed weight had significant positive correlations with oleic acid and stearic acid contents but was inversely associated with linoleic acid and palmitic acid contents. The increase in oleic acid content and decrease in the linoleic acid content were closely associated with increased 100-seed weight. Large variations in 100-seed weight, total oil content, and fatty acid profile were observed among the cultivar, breeding line, landrace, and unknown accession types. The cultivars exhibited the highest 100-seed weight, total oil content, and stearic acid and oleic acid contents, followed by the breeding line. However, the landraces had higher linoleic acid and palmitic acid contents than the breeding lines and cultivars. The large diversity among accession types provided evidence that could aid future studies of the domestication of peanuts. This study revealed parameters that could aid the selection of peanut seeds with health benefits and contribute to the development of new varieties containing high levels of oleic acid. Nevertheless, there are still many health-promoting nutrients needing to be well studied, such as vitamins, minerals, folic acid, niacin, bioactive polyphenolics, antioxidants, and so on. Further studies will be needed to investigate such beneficial nutrients, to provide a comprehensive knowledge for the breeding programs, consumers, and food industries.

## Figures and Tables

**Table 1:** Summary statistics of seed weight, total oil content, and individual and total fatty acid contents of the seeds of 301 peanut accessions.

Parameters	Maximum	Minimum	Mean	SD	CV (%)
SW (g/100-seed)	124.94	27.61	64.17	21.05	32.80
TO (%)	64.55	44.59	49.63	2.69	5.42
PA (%)	14.06	7.41	10.68	1.32	12.36
SA (%)	7.22	1.78	3.63	0.91	25.07
OA (%)	63.35	40.17	50.86	5.65	11.11
LA (%)	44.49	21.66	33.35	4.85	14.54
AA (%)	2.42	0.93	1.48	0.23	15.54
OL (%)	2.87	0.90	1.58	0.40	25.32
SFA (%)	21.31	12.77	15.79	1.40	8.87
UFA (%)	87.23	78.69	84.21	1.40	1.67

SW = 100-seed weight, TO = total oil, PA = palmitic acid, SA = stearic acid, OA = oleic acid, LA = linoleic acid, AA = arachidic acid, OL = oleic acid to linoleic acid ratio, SFA = total saturated fatty acid, UFA = total unsaturated fatty acid, SD = standard deviation; CV = coefficient of variation.

**Table 2:** Seed weight, total oil content, and individual and total fatty acid contents of the cultivar (*n* = 17), breeding line (*n* = 151), landrace (*n* = 61), and unknown (*n* = 72) genotypes.

Parameters	Values	Cultivar	Breeding Line	Landrace	Unknown	*p*–Value
SW(g/100-seed)	Range	39.92–106.44	27.61–124.94	34.56–92.26	27.79–83.38	***
	Mean	74.79 ^a^	73.68 ^a^	61.58 ^b^	43.91 ^c^	
	CV (%)	30.06	25.95	25.30	26.83	
TO (%)	Range	46.97–64.20	44.59–63.15	45.18–53.30	45.48–64.55	***
	Mean	51.69 ^a^	49.83 ^b^	48.31 ^c^	49.85 ^b^	
	CV (%)	9.29	5.08	3.83	5.02	
PA (%)	Range	7.97–12.36	7.41–13.65	8.77–13.45	9.02–14.06	***
	Mean	9.96 ^c^	10.28 ^c^	10.94 ^b^	11.49 ^a^	
	CV (%)	11.95	12.55	10.51	9.31	
SA (%)	Range	2.23–6.27	1.78–7.22	2.25–5.52	1.90–5.23	*
	Mean	4.02 ^a^	3.71 ^ab^	3.57 ^b^	3.42 ^b^	
	CV (%)	25.87	26.15	20.73	24.27	
OA (%)	Range	47.32–62.25	40.78–63.35	40.32–59.95	40.17–61.78	***
	Mean	53.63 ^a^	52.48 ^ab^	50.9 ^b^	46.77 ^c^	
	CV (%)	7.05	9.60	10.39	11.72	
LA (%)	Range	21.66–35.97	22.54–43.60	25.84–41.99	24.47–44.49	***
	Mean	30.88 ^c^	32.05 ^bc^	33.09 ^b^	36.87 ^a^	
	CV (%)	10.78	14.26	12.39	12.42	
AA (%)	Range	1.20–1.86	0.93–2.42	1.08–1.87	0.98–1.94	NS
	Mean	1.51 ^a^	1.48 ^a^	1.50 ^a^	1.46 ^a^	
	CV (%)	11.26	16.89	12.67	15.07	
OL (%)	Range	1.36–2.87	0.94–2.74	0.96–2.32	0.90–2.53	***
	Mean	1.77 ^a^	1.69 ^ab^	1.58 ^b^	1.31 ^c^	
	CV (%)	20.34	22.49	22.15	28.24	
SFA (%)	Range	14.19–18.42	12.77–21.31	13.02–19.60	13.29–19.39	***
	Mean	15.49 ^b^	15.47 ^b^	16.00 ^ab^	16.36 ^a^	
	CV (%)	8.20	7.82	9.31	9.41	
UFA (%)	Range	81.58–85.81	78.69–87.23	80.40–86.98	80.61–86.71	***
	Mean	84.51 ^a^	84.53 ^a^	83.99 ^ab^	83.64 ^b^	
	CV (%)	1.50	1.43	1.77	1.84	

SW = 100-seed weight, TO = total oil, PA = palmitic acid, SA = stearic acid, OA = oleic acid, LA = linoleic acid, AA = arachidic acid, OL = oleic acid to linoleic acid ratio, SFA = total saturated fatty acid, UFA = total unsaturated fatty acid, CV = coefficient of variation. Values in the same row marked with different superscript letters are significantly different (*p* < 0.05). NS, *, *** represent no significant or significant at *p* < 0.05, 0.001, respectively.

**Table 3:** Pearson correlation coefficients of seed weight, total oil content, and individual and total fatty acid contents based on analysis of all the data.

	SW	TO	PA	SA	OA	LA	AA	OL	SFA
TO	0.11								
PA	−0.71 ***	−0.14 *							
SA	0.25 ***	0.19 ***	−0.31 ***						
OA	0.60 ***	0.11	−0.80 ***	0.15 *					
LA	−0.55 ***	−0.12 *	0.73 ***	−0.30 ***	−0.98 ***				
AA	–0.04	0.01	−0.20 ***	0.57 ***	0.03	−0.13 *			
OL	0.58 ***	0.16 **	−0.76 ***	0.25 ***	0.98 ***	−0.98 ***	0.09		
SFA	−0.51 ***	−0.01	0.70 ***	0.44 ***	−0.65 ***	0.47 ***	0.34 ***	−0.54 ***	
UFA	0.51 ***	0.01	−0.70 ***	−0.44 ***	0.65 ***	−0.47 ***	−0.34 ***	0.54 ***	−1.00 ***

SW = 100-seed weight, TO = total oil, PA = palmitic acid, SA = stearic acid, OA = oleic acid, LA = linoleic acid, AA = arachidic acid, OL = oleic acid to linoleic acid ratio, SFA = total saturated fatty acid, UFA = total unsaturated fatty acid. *, **, *** represent significant at *p* < 0.05, 0.01, 0.001, respectively.

## Data Availability

Data are provided in the article and supplementary material.

## Supplementary Materials

The supporting information can be downloaded from https://www.mdpi.com/article/10.3390/foods11213463/s1. Table S1: Total oil content and individual and total fatty acid contents according to 100-seed weight; Table S2: Pearson correlation coefficients of seed weight, total oil content, and individual and total fatty acid contents among the cultivar, breeding line, landrace, and unknown accession types; Table S3: Principal component analysis of total oil, individual and total fatty acids of 301 peanut accessions, with eigenvalues and individual and cumulative contributions of variables in the first five principal components; Table S4: Average cluster values of the seed weight, total oil content, and individual and total fatty acid contents of 301 peanut accessions; Table S5: Peanut genotypes used in this study; Figure S1: Principal component biplots of the total oil content and individual and total fatty acid contents of the different 100-seed weight groups; Figure S2: Correlation coefficients of seed weight, total oil content, and individual and total fatty acid contents for the (A) cultivar (*n* = 17), (B) breeding line (*n* = 151), (C) landrace (*n* = 61), and (D) unknown (*n* = 72) accession types; Figure S3: Principal component analysis loading plots of targeted metabolites for the different 100-seed weight groups. (A) Group I, (B) Group II, (C) Group III, (D) Group IV, (E) Group V.
