# Supplementary material for: Seed Weight and Genotype Influence the Total Oil Content and Fatty Acid Composition of Peanut Seeds
Source: Foods. 2022 Nov 1;11(21):3463. doi: 10.3390/foods11213463 (PMC9653625; doi:10.3390/foods11213463)
Supplement: Supplementary file 1 [file foods-11-03463-s001.zip › foods-1994725-supplementary.pdf]

Table S1. Total oil content and individual and total fatty acid contents according to 100-seed weight.

Parameters	Values	Group I	Group II	Group III	Group IV	Group V	*P*-value
TO (%)	Range	45.48 − 64.55	44.59 − 53.55	45.18 − 58.68	44.90 − 63.15	49.63 − 64.20	***
	Mean	49.67^{b}	48.99^{b}	49.32^{b}	50.50^{b}	53.91^{a}	
	CV (%)	5.15	4.06	4.58	6.73	10.94	
PA (%)	Range	9.81 − 14.06	8.77 − 12.96	7.41 − 12.57	8.21 − 12.23	7.97 − 9.35	***
	Mean	12.02^{a}	10.83^{b}	10.10^{c}	9.68^{c}	8.67^{d}	
	CV (%)	7.74	9.33	8.81	9.71	6.11	
SA (%)	Range	1.88 − 7.22	1.78 − 6.23	1.98 − 5.97	2.54 − 5.82	4.07 − 6.27	***
	Mean	3.56^{c}	3.26^{c}	3.56^{c}	4.13^{b}	5.09^{a}	
	CV (%)	25.84	27.3	22.75	19.37	16.5	
OA (%)	Range	40.32 − 61.78	40.40 − 56.61	40.17 − 63.35	43.05 − 61.88	53.31 − 62.25	***
	Mean	45.86^{d}	50.3^{c}	$53.16b^{c}$	54.51^{ab}	56.45^{a}	
	CV (%)	9.09	9.72	8.95	7.47	6.24	
LA (%)	Range	24.47 − 43.75	25.71 − 43.60	23.89 − 44.49	22.54 − 40.88	21.66 − 31.62	***
	Mean	37.07^{a}	34.17^{b}	31.67^{bc}	30.25^{cd}	28.23^{d}	
	CV (%)	9.50	12.91	14.02	12.43	13.67	
AA (%)	Range	0.93 − 2.34	1.08 − 2.22	1.14 − 2.42	1.03 − 1.86	1.36 − 1.86	·
	Mean	1.49^{a}	1.43^{a}	1.51^{a}	1.42^{a}	1.57^{a}	
	CV (%)	16.11	16.08	15.23	10.56	12.10	
OL (%)	Range	0.93 − 2.53	0.93 − 2.20	0.90 − 2.65	1.05 − 2.74	1.69 − 2.87	***
	Mean	1.26^{d}	1.51^{c}	1.73^{b}	1.84^{ab}	2.05^{a}	
	CV (%)	20.63	21.19	20.81	19.02	22.93	
SFA (%)	Range	13.29 − 21.31	13.02 − 19.6	12.77 − 17.77	13.69 − 17.11	14.78 − 16.1	***
	Mean	17.07^{a}	15.52^{b}	15.17^{b}	15.23^{b}	15.32^{b}	
	CV (%)	8.67	7.86	6.00	5.38	3.66	
UFA (%)	Range	78.69 − 86.71	80.40 − 86.98	82.23 − 87.23	82.89 − 86.31	83.90 − 85.22	***
	Mean	82.93^{b}	84.48^{a}	84.83^{a}	84.77^{a}	84.68^{a}	
	CV (%)	1.78	1.44	1.07	0.97	0.67	

TO = total oil, PA = palmitic acid, SA = stearic acid, OA = oleic acid, LA = linoleic acid, AA = arachidic acid, OL = oleic acid to linoleic acid ratio, SFA = total saturated fatty acid, UFA = total unsaturated fatty acid, CV = coefficient of variation. Group I, Group II, Group III, Group IV, and Group V represent the genotypes have a 100-seed weight of 25–45 g, 45–65 g, 65–85 g, 85–105 g, and 105–125 g, respectively. Values in the same row with different superscript letters are significantly different ($p < 0.05$). ·, *** represent significant at $p < 0.1$, 0.001, respectively.

Table S2. Pearson correlation coefficients of seed weight, total oil content, and individual and total fatty acid contents among the cultivar, breeding line, landrace, and unknown accession types.

		**TO**	**PA**	**SA**	**OA**	**LA**	**AA**	**OL**	**SFA**	**UFA**
Cultivar (*n* = 17)	PA	-0.45								
	SA	0.59*	-0.43							
	OA	0.48	-0.85***	0.35						
	LA	-0.58*	0.75***	-0.59*	-0.94***					
	AA	0.35	-0.16	0.60*	0.06	-0.24				
	OL	0.61**	-0.76***	0.54*	0.95***	-0.99***	0.26			
	SFA	0.11	0.56*	0.50*	-0.50*	0.19	0.47	-0.23		
	UFA	-0.11	-0.56*	-0.50*	0.50*	-0.19	-0.47	0.23	-1.00***	
	SW	0.54*	-0.77***	0.49*	0.63**	-0.6*	0.09	0.59*	-0.31	0.31
Breeding line (*n* = 151)	PA	-0.21**								
	SA	0.15	-0.52***							
	OA	0.17*	-0.75***	0.36***						
	LA	-0.16*	0.67***	-0.50***	-0.97***					
	AA	0.05	-0.30***	0.58***	0.10	-0.20*				
	OL	0.19*	-0.71***	0.46***	0.98***	-0.98***	0.19*			
	SFA	-0.09	0.59***	0.37***	-0.49***	0.27***	0.36***	-0.35***		
	UFA	0.09	-0.59***	-0.37***	0.49***	-0.27***	-0.36***	0.35***	-1.00***	
	SW	0.09	-0.67***	0.36***	0.48***	-0.41***	-0.04	0.45***	-0.43***	0.43***
Landrace (*n* = 61)	PA	0.24								
	SA	-0.15	0.08							
	OA	-0.12	-0.80***	-0.38**						
	LA	0.12	0.74***	0.26*	-0.98***					
	AA	-0.17	-0.07	0.64***	-0.21	0.13				
	OL	-0.07	-0.74***	-0.32*	0.98***	-0.99***	-0.17			
	SFA	0.09	0.81***	0.64***	-0.84***	0.72***	0.40**	-0.76***		
	UFA	-0.10	-0.81***	-0.64***	0.85***	-0.73***	-0.40**	0.76***	-1.00***	
	SW	-0.17	-0.67***	-0.19	0.61***	-0.55***	-0.14	0.57***	-0.63***	0.64***
Unknown (*n* = 72)	PA	0.05								
	SA	0.20	0.20							
	OA	0.01	-0.79***	-0.23*						
	LA	-0.06	0.68***	0.03	-0.97***					
	AA	-0.09	-0.07	0.49***	-0.04	-0.07				
	OL	0.05	-0.73***	-0.16	0.98***	-0.97***	-0.03			
	SFA	0.13	0.79***	0.74***	-0.68***	0.48***	0.36**	-0.60***		
	UFA	-0.13	-0.79***	-0.74***	0.68***	-0.48***	-0.36**	0.60***	-1.00***	
	SW	0.00	-0.46***	-0.17	0.39***	-0.32**	-0.22	0.40***	-0.44***	0.44***

SW = 100-seed weight, TO = total oil, PA = palmitic acid, SA = stearic acid, OA = oleic acid, LA = linoleic acid, AA = arachidic acid, OL = oleic acid to linoleic acid ratio, SFA = total saturated fatty acid, UFA = total unsaturated fatty acid. *, **, *** represent significant at $p < 0.05, 0.01, 0.001$, respectively.

Table S3. Principal component analysis of total oil, individual and total fatty acids of 301 peanut accessions, with eigenvalues and individual and cumulative contributions of variables in the first five principal components.

**Parameters**	**PC1**	**PC2**	**PC3**	**PC4**	**PC5**
TO	0.40	2.11	92.35	2.14	2.88
PA	16.97	1.53	0.14	16.88	15.95
SA	0.07	36.97	0.04	0.01	39.96
OA	19.86	0.79	0.21	5.94	2.21
LA	16.93	4.29	0.26	13.68	3.61
AA	0.02	25.81	6.99	34.21	31.54
OL	18.17	2.82	0.01	9.42	3.20
SFA	13.78	12.84	0.00	8.88	0.32
UFA	13.79	12.83	0.00	8.84	0.33
Eigenvalue	4.69	2.30	0.98	0.65	0.37
Variability (%)	52.09	25.53	10.93	7.19	4.08
Cumulative (%)	52.09	77.62	88.54	95.74	99.82

TO = total oil, PA = palmitic acid, SA = stearic acid, OA = oleic acid, LA = linoleic acid, AA = arachidic acid, OL = oleic acid to linoleic acid ratio, SFA = total saturated fatty acid, UFA = total unsaturated fatty acid.

Table S4. Average cluster values of the seed weight, total oil content, and individual and total fatty acid contents of 301 peanut accessions.

**Cluster**	**No. Acc.**	**SW**	**TO**	**PA**	**SA**	**OA**
I	120	47.48 ± 16.31^{c}	49.74 ± 2.63^{b}	11.92 ± 0.90^{a}	3.59 ± 0.87^{b}	44.99 ± 3.12^{c}
II	75	65.48 ± 12.90^{b}	48.22 ± 1.78^{c}	10.42 ± 0.49^{b}	2.92 ± 0.39^{c}	53.49 ± 2.87^{b}
III	106	82.14 ± 14.20^{a}	50.52 ± 2.89^{a}	9.46 ± 0.73^{c}	4.19 ± 0.84^{a}	55.64 ± 2.67^{a}
P-value		***	*	***	***	***
Cluster	LA	AA	OL	SFA	UFA	
I	38.01 ± 3.12^{a}	1.48 ± 0.23^{ab}	1.20 ± 0.19^{c}	16.99 ± 1.20^{a}	83.01 ± 1.20^{c}	
II	31.74 ± 2.85^{b}	1.43 ± 0.17^{b}	1.71 ± 0.25^{b}	14.77 ± 0.65^{c}	85.23 ± 0.65^{a}	
III	29.21 ± 2.56^{c}	1.51 ± 0.25^{a}	1.93 ± 0.27^{a}	15.15 ± 0.94^{b}	84.85 ± 0.94^{b}	
P-value	***	NS	***	***	***	

SW = 100-seed weight, TO = total oil, PA = palmitic acid, SA = stearic acid, OA = oleic acid, LA = linoleic acid, AA = arachidic acid, OL = oleic acid to linoleic acid ratio, SFA = total saturated fatty acid, UFA = total unsaturated fatty acid. Values in the same row with different superscript letters are significantly different ($p < 0.05$). NS, *, *** represent not significant or significant at $p < 0.05$, 0.001, respectively.

Table S5. Peanut genotypes used in this study.

No	Genotype	No	Genotype	No	Genotype	No	Genotype
1	IT30815	76	IT172536	151	IT191622	226	IT30881
2	IT30842	77	IT172538	152	IT191625	227	IT30885
3	IT30844	78	IT172540	153	IT191626	228	IT30915
4	IT30854	79	IT172541	154	IT191627	229	IT30917
5	IT30863	80	IT172542	155	IT191628	230	IT30918
6	IT30919	81	IT172543	156	IT191629	231	IT30920
7	IT30929	82	IT172545	157	IT191630	232	IT30921
8	IT30960	83	IT172548	158	IT191631	233	IT30922
9	IT30962	84	IT172553	159	IT194509	234	IT30925
10	IT101139	85	IT172554	160	IT196366	235	IT30932
11	IT101146	86	IT172556	161	IT203648	236	IT30935
12	IT101172	87	IT172562	162	IT203650	237	IT30956
13	IT101184	88	IT172572	163	IT203652	238	IT101182
14	IT101204	89	IT172600	164	IT207978	239	IT103386
15	IT101210	90	IT172610	165	IT209224	240	IT103417
16	IT101216	91	IT172626	166	IT212142	241	IT103961
17	IT101229	92	IT172628	167	IT212145	242	IT105693
18	IT108816	93	IT172642	168	IT212146	243	IT110215
19	IT110209	94	IT172657	169	IT212147	244	IT110216
20	IT110210	95	IT172658	170	IT212148	245	IT110220
21	IT110211	96	IT172670	171	IT212149	246	IT110236
22	IT110212	97	IT172672	172	IT212150	247	IT110957
23	IT110218	98	IT172673	173	IT212151	248	IT112892
24	IT110219	99	IT172681	174	IT212152	249	IT113440
25	IT110221	100	IT172683	175	IT212153	250	IT172452
26	IT110223	101	IT172684	176	IT212154	251	IT172539
27	IT110234	102	IT172693	177	IT212155	252	IT172555
28	IT110235	103	IT172694	178	IT212156	253	IT172659
29	IT110237	104	IT172695	179	IT212157	254	IT172667
30	IT110238	105	IT172696	180	IT212158	255	IT172787
31	IT110240	106	IT172700	181	IT212159	256	IT172815
32	IT110241	107	IT172702	182	IT212160	257	IT172817
33	IT110242	108	IT172705	183	IT212161	258	IT172818
34	IT110244	109	IT172710	184	IT212162	259	IT172821
35	IT110245	110	IT172711	185	IT212163	260	IT172822
36	IT110246	111	IT172715	186	IT212164	261	IT172826
37	IT121450	112	IT172716	187	IT212165	262	IT172827
38	IT144017	113	IT172718	188	IT212166	263	IT172828
39	IT171372	114	IT172726	189	IT212179	264	IT181793
40	IT171373	115	IT172729	190	IT212181	265	IT181796
41	IT171374	116	IT172730	191	IT212194	266	IT181800
42	IT171376	117	IT172738	192	IT212198	267	IT181803
43	IT171377	118	IT172739	193	IT212203	268	IT181805
44	IT172392	119	IT172743	194	IT212204	269	IT181809
45	IT172393	120	IT172757	195	IT184834	270	IT181815
46	IT172394	121	IT172820	196	IT184884	271	IT181816
47	IT172395	122	IT172825	197	IT184907	272	IT181818
48	IT172397	123	IT175811	198	IT184917	273	IT181820
49	IT172398	124	IT181765	199	IT184929	274	IT181821
50	IT172399	125	IT181766	200	IT318096	275	IT181822
51	IT172402	126	IT181771	201	IT318097	276	IT181823
52	IT172403	127	IT181774	202	IT318802	277	IT181825

53	IT172405	128	IT181775	203	IT318803	278	IT181826
54	IT172407	129	IT181777	204	IT318804	279	IT181827
55	IT172408	130	IT181789	205	IT318805	280	IT181830
56	IT172413	131	IT181791	206	IT318806	281	IT181833
57	IT172418	132	IT181795	207	IT318807	282	IT181834
58	IT172419	133	IT185464	208	IT318808	283	IT181837
59	IT172420	134	IT185678	209	IT318809	284	IT181838
60	IT172421	135	IT185682	210	IT318810	285	IT184826
61	IT172422	136	IT191602	211	IT318811	286	IT185676
62	IT172423	137	IT191603	212	IT321093	287	IT185679
63	IT172426	138	IT191604	213	IT321094	288	IT185680
64	IT172427	139	IT191605	214	IT334404	289	IT185681
65	IT172431	140	IT191606	215	IT334405	290	IT201361
66	IT172458	141	IT191607	216	IT334406	291	IT201362
67	IT172467	142	IT191608	217	IT334407	292	IT201363
68	IT172469	143	IT191609	218	IT334408	293	IT201364
69	IT172479	144	IT191610	219	IT334409	294	IT201370
70	IT172484	145	IT191613	220	IT30864	295	IT201371
71	IT172502	146	IT191614	221	IT30871	296	IT202680
72	IT172507	147	IT191615	222	IT30872	297	IT208799
73	IT172508	148	IT191619	223	IT30873	298	IT210195
74	IT172517	149	IT191620	224	IT30878	299	IT210196
75	IT172528	150	IT191621	225	IT30879	300	IT226447
301	IT250592						

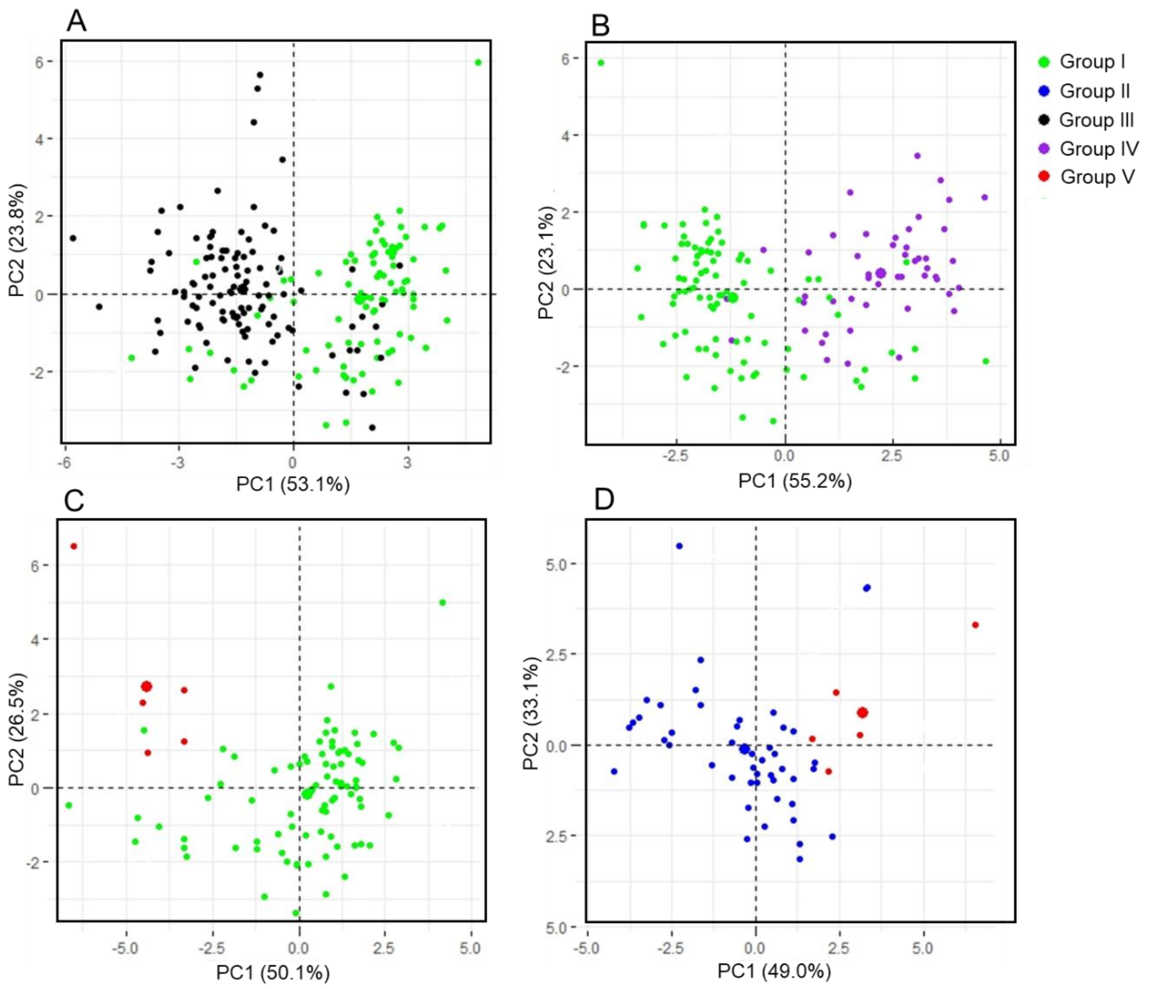

Figure S1. Principal component biplots of the total oil content and individual and total fatty acid contents of the different 100-seed weight groups. Group I, Group II, Group III, Group IV, and Group V represent the genotypes have a 100-seed weight of 25–45 g, 45–65 g, 65–85 g, 85–105 g, and 105–125 g, respectively. (A) Loading plots of Groups I and III, (B) Loading plots of Groups I and IV, (C) Loading plots of Groups I and V, (D) Loading plots of Groups II and V.

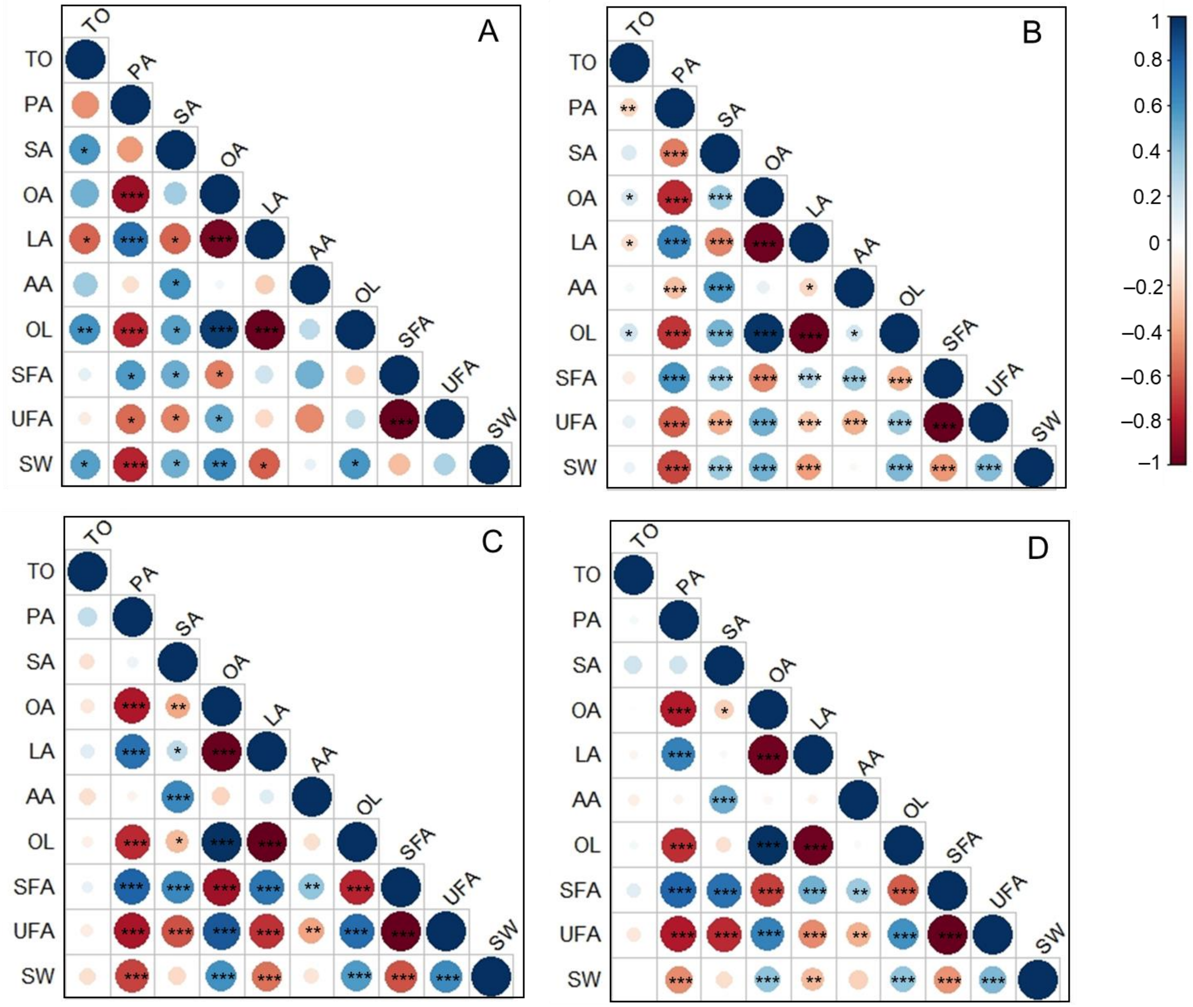

Figure S2. Correlation coefficients of seed weight, total oil content, and individual and total fatty acid contents for the (A) cultivar (n = 17), (B) breeding line (n = 151), (C) landrace (n = 61), and (D) unknown (n = 72) accession types. TO = total oil, PA = palmitic acid, SA = stearic acid, OA = oleic acid, LA = linoleic acid, AA = arachidic acid, OL = oleic acid to linoleic acid ratio, SFA = total saturated fatty acid, UFA = total unsaturated fatty acid, SW = 100-seed weight. *, **, *** represent significant at $p < 0.05$, 0.01, 0.001, respectively.

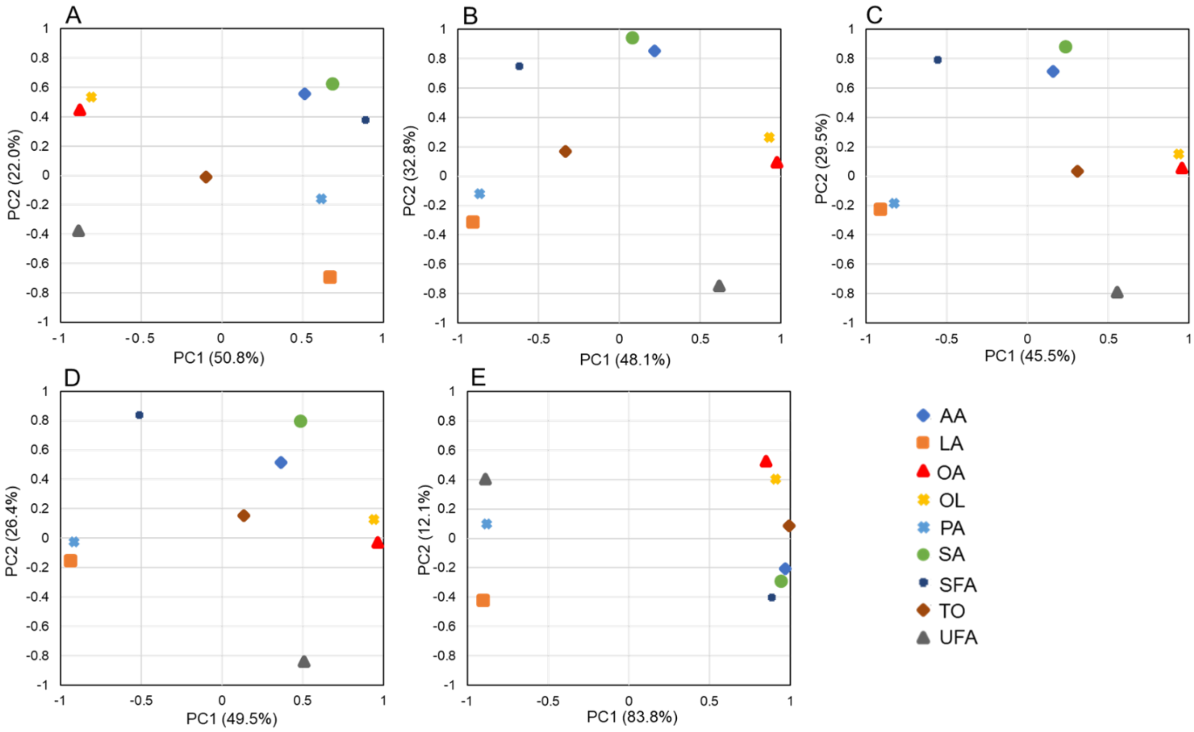

Figure S3. Principal component analysis loading plots of targeted metabolites for the different 100-seed weight groups. (A) Group I, (B) Group II, (C) Group III, (D) Group IV, (E) Group V. Group I, Group II, Group III, Group IV, and Group V represent the genotypes have a 100-seed weight of 25–45 g, 45–65 g, 65–85 g, 85–105 g, and 105–125 g, respectively. TO = total oil, PA = palmitic acid, SA = stearic acid, OA = oleic acid, LA = linoleic acid, AA = arachidic acid, OL = oleic acid to linoleic acid ratio, SFA = total saturated fatty acid, UFA = total unsaturated fatty acid.
